# Dependence of connectivity on geometric distance in brain networks

**DOI:** 10.1038/s41598-019-50106-2

**Published:** 2019-09-16

**Authors:** Alessio Perinelli, Davide Tabarelli, Carlo Miniussi, Leonardo Ricci

**Affiliations:** 1 Department of Physics, University of Trento, 38123 Trento, Italy; 2 CIMeC, Center for Mind/Brain Sciences, University of Trento, 38068 Rovereto, Italy

**Keywords:** Neuroscience, Biological physics

## Abstract

In any network, the dependence of connectivity on physical distance between nodes is a direct consequence of trade-off mechanisms between costs of establishing and sustaining links, processing rates, propagation speed of signals between nodes. Despite its universality, there are still few studies addressing this issue. Here we apply a recently–developed method to infer links between nodes, and possibly subnetwork structures, to determine connectivity strength as a function of physical distance between nodes. The model system we investigate is brain activity reconstructed on the cortex out of magnetoencephalography recordings sampled on a set of healthy subjects in resting state. We found that the dependence of the time scale of observability of a link on its geometric length follows a power–law characterized by an exponent whose extent is inversely proportional to connectivity. Our method provides a new tool to highlight and investigate networks in neuroscience.

## Introduction

The fact that the brain has a small–world topology makes up a widespread assumption regarding the investigation of brain connectivity^1–3^. The issue is linked to the question of how geometric distance is relevant in human brain networks. As pointed out by Bullmore and Sporns in their review of 2012^1^, the dependence of connectivity on the physical distance appears to be a trade-off between the complexity required to carry out cognitive tasks and the metabolic costs of establishing and sustaining a huge number of elements and links. More recently, Gollo *et al*.^4^ investigated the balance between costs of anatomical wirings and complexity, suggesting that any perturbation might induce neuropsychiatric disorders. While neural connections within the brain are not straight segments, geometric (Euclidean) distance turns out to be a measure apt to describe distance–related issues concerning the brain function^5,6^. Geometric distance between network elements was shown to be a relevant parameter for modelling the information transfer between brain regions, e.g. with regard to information transfer delays^7,8^. The interplay between geometric distance, connectivity and network topology was also investigated in relation to neurological diseases^9^. The dependence of functional links on the geometric distance between brain regions was first investigated by Salvador *et al*.^10,11^ and Fair *et al*.^12^ by studying correlation coefficients assessed out of functional magnetic resonance imaging (fMRI) recordings. In these works, as well as in more recent ones^1,9^, correlation coefficients are proposed to be proportional to the inverse of the square distance. On the other hand, other studies hint at different dependencies of connectivity on distance^13–15^. For example, Expert *et al*.^16^ claimed that, for sufficiently small ranges, correlations decrease as the inverse of the square root of distance.

In this paper we investigate the dependence on distance of the basic elements of networks, i.e. links between pairs of nodes. Links are assessed by means of a recently introduced method^17^ that, rather than relying on standard correlation measures, looks at the time scale at which cross–correlation between reciprocally undelayed time series occurs. The new approach is therefore complementary to traditional tools used to assess connectivity, and is expected to provide alternative insights into this issue.

We used cortical activity reconstructed out of magnetoencephalography (MEG) resting state time series collected from 20 healthy subjects among those available in the Human Connectome Project database^18,19^. A set of 72 brain regions, henceforth referred to as nodes, was randomly selected. Given a pair of nodes, we then investigated the dependence on their distance, henceforth referred to as link length *d*, of the time scale *W* at which cross–correlation between time series generated by each node occurs. This dependence turned out to be consistent with a power–law.

## Results: Time Scale of Observability vs. Distance

The reconstruction of the cortical activity was carried out according to the atlas by Glasser *et al*.^20^, which provides the position of 360 different nodes. For the sake of computational simplicity, we analyzed 1/5 of the available set, namely 72 randomly selected nodes. For each single node a set of 60 time series is available, corresponding to 20 subjects and 3 recordings per subject. The set of nodes results in 2556 pairs. Due to the slight anatomical differences between the subjects, each pair corresponds to a set of 20 link lengths, so that the total number of *d* values is approximately 50000 within the range from 5 to 160 mm.

Given a subject, a recording and a pair of nodes, the time scale of observability of the corresponding link was assessed out of the related pair of time series, resulting in approximately 150000 values of *W*. While ∼ 70000 assessments failed to produce a finite time scale *W*, the remaining ∼ 83000 ones provided a valid *W* value within the range from 0.4 s to 48 s.

**Figure 1:**
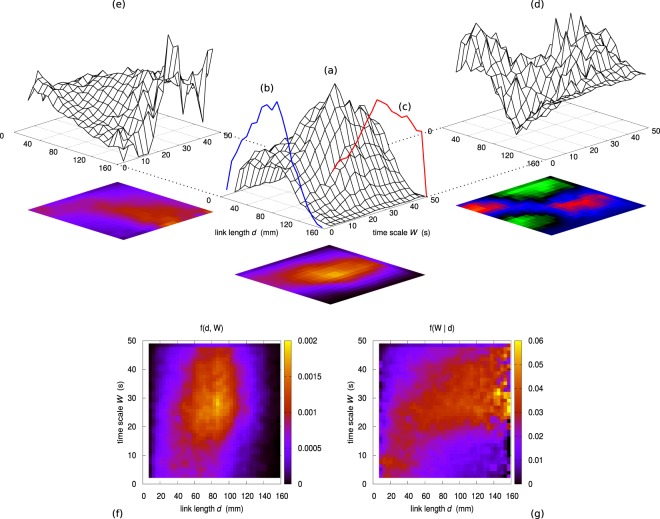
(**a**) Joint sample probability distribution $f(d,W)$. Both the distance range and the time scale range are partitioned in 20 bins each. Blue line (**b**) Marginal sample probability distribution of *d*. Red line (**c**) Marginal sample probability distribution of *W*. (**d**) Difference between the joint distribution $f(d,W)$ and the product ${g}_{d}(d)\cdot {g}_{W}(W)$ of the two marginal distributions. (**e**) Conditional sample distribution $f(W|d)$ of *W* given *d*, evaluated as $f(d,W)/{g}_{d}(d)$. (**f**) Map representation of the joint sample probability distribution $f(d,W)$ obtained by partitioning both the distance range and the time scale range in 40 bins. (**g**) Map representation of the conditional sample probability distribution $f(W|d)$ obtained by partitioning both the distance range and the time scale range in 40 bins.

The first goal of the present work is to verify whether there is a correlation between link length *d* and time scale of observability *W*. Figure 1(a) shows the joint sample probability distribution $f(d,W)$ obtained by partitioning both the distance range and the time scale range in 20 bins each. The two marginal distributions ${g}_{d}(d)$ and ${g}_{W}(W)$ are shown in Fig. 1(b,c), respectively. Figure 1(d) shows the difference $f(d,W)-{g}_{d}(d)\cdot {g}_{W}(W)$, which turns out to be significantly nonzero. Consequently, the two variables *d* and *W* turn out to be significantly correlated. Besides in the (a) part of the figure, the color map representing $f(d,W)$ is shown in Fig. 1(f) where the bin size is reduced by a factor 2 on each direction. The slight asymmetry of the shape hints as well at a correlation between *d* and *W*.

**Figure 2:**
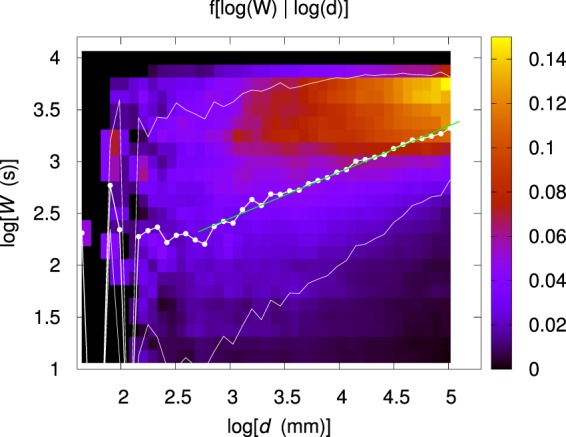
Map representation of the conditional sample probability distribution $f[\,\log (W)|\,\log (d)]$ obtained by partitioning both the distance range and the time scale range in 40 bins. The white dots represent the average value $\overline{W}$ of *W* given *d*, while the upper and lower white, thin lines bound the 68% confidence region for *W*. The green straight line corresponds to the best linear fit to the average points for which $d\geqslant 15\,{\rm{mm}}$. The slope corresponds to $\gamma =0.44\pm 0.01$.

Figure 1(e) shows the conditional sample distribution $f(W|d)=f(d,W)/{g}_{d}(d)$. The same distribution, upon halving again the bin size on each direction, is shown in Fig. 1(g). The shape of the most likely region suggests that the relationship between *W* and *d* is nonlinear. As explained in the Methods section, among different functional forms analyzed, a power–law of the kind $W={W}_{0}{(\frac{d}{{d}_{0}})}^{\gamma }$ suitably describes the dependence of *W* on *d*. The result of this analysis is shown in Fig. 2. Upon setting the normalization parameter *d*_0_ to 75 mm (see Methods section), the parameters *W*_0_ and *γ* resulting from a best–fit procedure are ${W}_{0}=(20.9\pm 0.2)\,{\rm{s}}$ and $\gamma =0.44\pm 0.01$.

**Figure 3:**
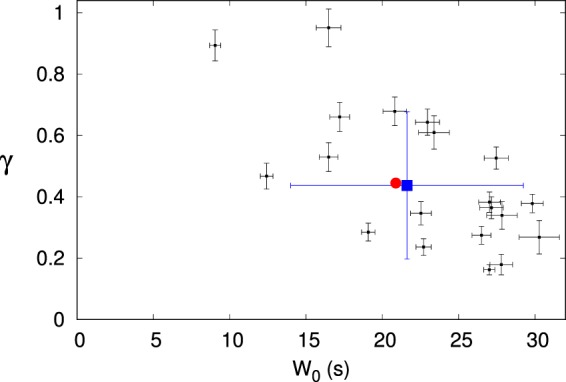
Cartesian representation of the $({W}_{0},\gamma )$ pairs resulting from the power–law fit on each single subject (black dots) and on the whole set of subjects (red dot). The errorbars correspond to the uncertainties on the fit parameters; in the case of the whole set of subjects, errorbars are too small to display. The blue dot and the related errorbars correspond to the sample mean and sample standard deviation of the coordinates *W*_0_ and *γ* of the 20, single–subject black dots.

The same analysis explained above was applied to each single subject in order to test whether the previous behaviour is characteristic of a single human brain or, rather, is the spurious effect of a cohort analysis. The results are shown in Fig. 3. The power–law dependence of *W* on *d* is indeed present in each subject, although with different values of the parameters *W*_0_ and *γ*. Most parameters pairs are clustered in a region where *W* ranges from 15 mm to 30 mm and *γ* ranges from 0.2 to 0.7. This result is in agreement with the claim by Expert *et al*.^16^. As far as the exponent *γ* is concerned, a possible explanation of its variability relies on different levels of connectivity, as discussed in the next section. Interestingly, the average subject behaviour, in terms of average values of the two parameters among the subjects (blue dot), is in a very good agreement with the behaviour extrapolated by a pooled analysis of all subjects (red dot).

## Discussion

As asserted in the Introduction, due to the presence of physical constraints, connectivity has to depend on distance^21^. This property appears to be universal, i.e. independent of the system under investigation. As an example, parallel to the work by Bullmore and Sporns^1^ concerning trade-off issues in brain connectivity, the work by Gastner and Newman^22^ addresses distributions of geometric properties in terms of costs and benefits within the framework of geographical networks. However, despite its universality, studies addressing the dependence of connectivity on physical distance are still few. Among these ones, a recent work by Hens *et al*.^23^ discusses a general model for signal propagation in networks to classify them in families depending on “the interplay between network paths, degree distribution and interaction dynamics”.

In neuroscience, to separate local–scale and large–scale regimes, Bellec *et al*.^24^ described distance–dependent correlation between fMRI time series by relying on variograms, a tool from spatial statistics to quantify correlation as a function of distance^25^. Variograms allowed to empirically extract information on the spatial extent of correlations in fMRI connectivity^26–28^ and to account for *the complicated characteristics of fMRI data*^29^. In addition, variograms were applied to remove spurious correlations due to voxel proximity in fMRI studies within the auditory cortex^30^ and to monitor the spatial distribution of cellular activity in the brain^31^. In general, correlation is shown to quickly decrease down to a critical distance and then to saturate. A crucial issue in the investigation of human brain connectivity is to establish whether, and to what extent, structural connectivity, assessed by diffusion tensor imaging and tractography^32^, determines functional connectivity^33–36^. This issue is ultimately linked to how the neuron wiring is related to brain cognitive functions^4,37^ and how it is possible to reconstruct physical links out of temporal correlations detected through electrophysiological measurements.

Our investigation tackles the problem of determining the dependence of the connectivity strength on the geometric distance in a link between two nodes. Connectivity strength is expressed in terms of time scale of observability assessed by exploiting an analytical tool recently developed that relies on the analysis of time series each stemming from a single node. In the present case, time series are cortical activities reconstructed out of MEG recordings.

What we observe, by using an approach based on the analysis of distributions similar to that discussed by Bialek *et al*.^38^, is that the dependence appears to be a power–law of the kind *W* ∼ *d*^*γ*^, where *W* is the time scale of observability and *d* is the geometric distance. The exponent *γ* takes on values ranging from 0.2 to 0.7. Lower values of *γ* corresponds to higher levels of connectivity, as explained in the following.

The quantity *W* measures the time scale at which the cross–correlation between time series generated by two nodes becomes significantly visible. The source of cross–correlation are typically peak–like^17^ events that occur in both nodes at the same time. This process is countered by noise, which tends to wash out cross–correlation. If nodes are directly connected by physical links, and if the propagation speed of signals between nodes is much faster than the time scale of observability of cross–correlation – as it is the case of neural signals, which propagate in ms, whereas *W* is at least of order 1 s – the time scale of observability of links is not expected to depend on their length. In this case, *γ* is expected to be ∼ 0. On the other hand, if no direct link between two nodes exists, a working link has to rely on intemediate nodes that act as relay hubs. The relay process possibly introduces a noise component, which leads to a progressive signal degradation as distance increases. Consequently, the larger the distance *d*, the less frequent a peak–like co–activating process occurs, and thus the longer is the time window *W* required to observe peak–like events that occur at the same time.

On the basis of the large variety of neural connections occurring in the human brain, it is possible that different mechanisms like the two ones mentioned above simultaneously contribute to the observed power–law behaviour. In addition, different behaviours can be expected if particular sets of nodes, for example making up a subnetwork (like the default mode network^39,40^), are considered, as well as if correlation between the activity of nodes is not due to a direct information link but it is rather the manifestation of simultaneous responses to a common stimulation. The approach presented in this paper can be used to identify sets of nodes that form a subnetwork, for example by characterizing them on the basis of a specific behaviour in the $({W}_{0},\gamma )$ parameter space.

The exact identification and quantification of these mechanisms is beyond the scope of the present work. One possible way of tackling this issue is to study the complexity of neurophysiological signals^41^ and its influence on the time scale of observability when pairs of signals are analyzed. This approach requires analytical techniques and observables typical of nonlinear time series analysis like embedding^42,43^, correlation dimension^44^, maximum Lyapunov exponent^45,46^ and permutation entropy^47^.

In conclusion, we found that the the link strength, in terms of time scale of observability, significantly depends on the geometric link length. The method discussed in this work can be used to highlight the presence of an underlying subnetwork structure between subsets of nodes.

## Methods

### Observability of a link inferred out of zero–delay cross–correlation analysis of the constituent nodes

In this work the assessment of connectivity between brain regions is carried out by applying a recently–introduced zero–delay cross–correlation method^17^. The aim of the algorithm^48^ is to assess the existence of *links* between nodes of a possible subnetwork structure out of time series recorded at each node and to provide an estimate of the time scale on which an existing link is observable.

**Figure 4:**
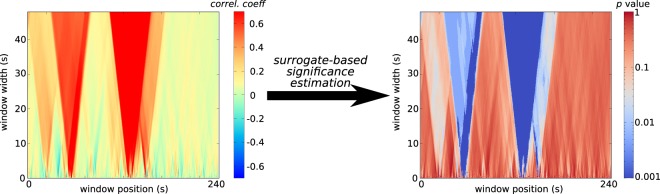
Correlation diagram (left) and *p*–value diagram (right) for the R–TF and the R–s32 brain regions (see Table 1) computed on one recording of the second subject.

The input of the analysis is a pair of time series, each associated to one of the two nodes. The first step to provide an evidence of a link between the two nodes consists of evaluating the zero–delay cross–correlation between the two time series. Cross–correlation is computed as the sample Pearson correlation coefficient over moving time windows of different widths. Therefore, correlation coefficients turn out to depend on both the window position and width, as displayed by means of two–dimensional correlation diagrams shown in Fig. 4 (left). In the present work, the window width was set to span a time interval from 400 ms to 48 s.

To assess the significance of the correlation coefficients, i.e. to associate a *p*–value to each correlation coefficient, a surrogate–based approach is followed^49^. Surrogate time series are generated according to an iterative algorithm that preserves both the distribution of amplitudes and, approximately, the autocorrelation function of the original sequence. Given the pair of original time series, a set of 200 pairs of surrogate time series are generated. For each surrogate pair, a correlation coefficient diagram similar to the one shown in Fig. 4 is computed. The *p*–value of the point corresponding to a given window width and window position is then computed by ranking the correlation coefficient of the original time series within the set of 200 surrogate values, and finally normalizing the rank by 200. For each pair of nodes, the analysis provides a *p*–value diagram that depends on the window position and width. Figure 4 (right) shows the *p*–value diagram corresponding to the correlation diagram displayed in Fig. 4 (left).

A *p*–value diagram is then further processed to assess the existence of a link between the two nodes. This step requires the evaluation of the *efficiency η* corresponding to a window width *w*, i.e. of the function *η* = *η*(*w*): given the *p*–value diagram for the pair of nodes, and given a value *w* of the window width, the efficiency *η*(*w*) is defined as the fraction of the running windows of width *w* that exhibit a *p*–value smaller than a given significance threshold. In this work, this significance threshold is set to 5%. Efficiency is typically a growing function of *w*^17^. A link between two nodes is deemed to exist at a time scale *W* if the efficiency at the window width *W* overcomes a second threshold that is here set to 0.5. If the efficiency fails to overcome the threshold, no link is attributed to the pair of nodes.

The window width *W* defines the minimum time scale at which a link starts to be observable: hereafter, the link is supposed to exist for any time scale larger than *W*, at least up to the maximum window width of 48 s. It should be noted that observability eventually fades out – so that the corresponding link disappears – once the observation window becomes so wide that the noisy contributions to the time series become dominant again^17^.

The time scale of observability of a link is a measure of the minimum observation window such that two nodes are deemed to be correlated. For example, in the case of two nodes showing an identical activity over time, the minimum time scale of observability is zero. More realistically, the activity of two nodes turns out to be co–activated only for short periods of time, for example if a subnetwork structure is established for a given purpose and then reallocated after the purpose is accomplished. In this case, correlation is observed only if a sufficiently large window is used, and the time scale of observability turns out to correspond to the repetition time of this co–activation. The value *W* thus provides an estimate of the time scale of the process underlying the activation of links. These time scales are not necessarily related to the information transfer speed across the subnetwork: in the case of brain networks, information transfer occurs at the millisecond scale while the activation of links spans time intervals of second or tens of seconds^17,40,50^.

Given a number *N* of candidate nodes of a possible subnetwork structure, there are $N(N-\mathrm{1)/2}$ possible links, each corresponding to a node pair. The analysis described above has then to be carried on each of these pairs, and the results further processed in order to assess the possible presence of an underlying subnetwork structure^17^.

### Dataset and preprocessing

The dataset used in this work consists of MEG resting state recordings of 20 healthy subjects (age between 22 and 35, 16 males, 4 females) blindly extracted from the public database of the Human Connectome Project (HCP)^18,19^. The HCP provides the required ethical approval and consent needed for study and dissemination. Procedures for subject recruitment, including informed consent forms and consent to share de–identified data, were approved by the Institutional Review Board of the Washington University in St. Louis. All experimental procedures were performed under the guidelines of the HCP, which adhered to the relevant IRB processes related to that project.

In brief, for each subject, three MEG resting state sessions of about 5 minutes each are available. Data were recorded with participants lying in supine position in a whole–head 248 magnetometers MAGNES 3600 scanner (4D Neuroimaging, San Diego, CA). Participants were instructed to rest with open eyes and maintain fixation on a projected red crosshair on a dark background. MEG sensor data, sampled at 2035 Hz, were cleaned by excluding bad channels and other artifacts and removing ocular/cardiac/myogenic activity by means of independent component analysis. The public HCP database provides single–shell volume conduction models^51^ computed out of a brain–enclosing surface mesh with 5000 points, as well as surface reconstructions of the mid–thickness cortical mantle, both segmented from individual anatomical T1–weighted MRI scan (Siemens Trio 3 T - Siemens Healthcare GmbH, Erlangen, Germany). All meshes coordinates are standardized to the MNI space and co–registered to the sensor array. Further details can be found on the HPC website (MEG connectome pipeline version 3.0)^19^. Cortical activity was reconstructed by means of a minimum norm algorithm^52^ with unconstrained dipole orientations. We used an 8004 points cortical mesh as a source model, resulting in a grid resolution of approximately 5 mm. Noise covariance was estimated from the available empty–room recordings and no regularization was applied. Preprocessing and source reconstruction were carried out by means of FieldTrip routines^53^. After the reconstruction process, the time series were resampled from 2035 Hz down to 250 Hz. In order to get equally–long time series of 300 s duration (75000 samples), the first 4 seconds of each time series as well as a final segment of variable length were discarded.

Table 1 lists the 72 brain areas randomly selected out of the 360 areas defined in the atlas by Glasser *et al*.^20^, which was built by combining structural, diffusion, functional and resting state MRI data from 210 healthy young individuals. The random selection was carried out by the following procedure:

1. number the areas between 1 and 360;
2. toss a number between 1 and 360 by means of a uniform random number generator and thus select the first area;
3. toss another number *k* between 1 and 360;
4. check whether *k* is equal to anyone of the previously tossed numbers; if yes, repeat operation 3, otherwise jump to the next step;
5. check whether the new area lies within 1 cm of anyone of the previously selected areas; if yes, repeat operation 3, otherwise accept the new area and jump to the next step;
6. check whether the total number of areas is less than 72; if yes, repeat operation 3, otherwise stop.

**Table 1:** List of the 72 brain areas selected for the analysis. The reader can refer to *Supplementary Neuroanatomical Results* by Glasser *et al*.^20^ for anatomical and functional details about the areas listed here.

Nr.	Atlas area (hemisphere)	Nr.	Atlas area (hemisphere)	Nr.	Atlas area (hemisphere)
1	TF (right)	25	V4 (right)	49	TPOJ1 (right)
2	V3B (right)	26	52 (right)	50	PeEc (left)
3	AAIC (right)	27	IFJa (right)	51	11 l (right)
4	10pp (right)	28	TF (left)	52	8 C (right)
5	6r (right)	29	s6–8 (right)	53	MIP (left)
6	47 s (left)	30	V4 (left)	54	EC (left)
7	POS2 (right)	31	OP2–3 (right)	55	7AL (left)
8	s32 (right)	32	STGa (right)	56	24dv (right)
9	p24pr (left)	33	25 (right)	57	5m (left)
10	FOP2 (left)	34	VVC (left)	58	IFJp (left)
11	6ma (left)	35	a32pr (left)	59	V6A (right)
12	FOP4 (right)	36	10r (left)	60	23d (left)
13	STV (left)	37	p9–46v (left)	61	6 mp (right)
14	PFcm (left)	38	47 m (right)	62	STGa (left)
15	STSvp (left)	39	VVC (right)	63	7 m (left)
16	PeEc (right)	40	LIPd (right)	64	a10p (right)
17	PI (right)	41	H (left)	65	a47r (right)
18	OFC (left)	42	RSC (right)	66	AVI (left)
19	PBelt (right)	43	IFSa (right)	67	PFm (right)
20	p9–46v (right)	44	43 (right)	68	p10p (right)
21	TE1a (right)	45	45 (right)	69	7Pm (left)
22	31a (left)	46	V6 (right)	70	24dd (right)
23	FOP4 (left)	47	52 (left)	71	10r (right)
24	9–46d (left)	48	7PC (left)	72	s32 (left)

The MNI coordinates of the centroid of each area provide the locations for the 72 sources that identify the respective nodes.

**Figure 5:**
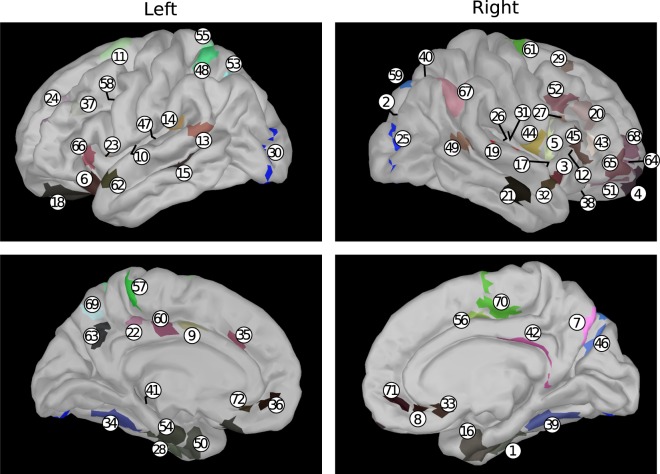
Anatomical position of the areas listed in Table 1 on a default anatomy. Colors are consistent with those used in the atlas by Glasser *et al*.^20^ and are related to the functional group to which each area belongs.

Figure 5 shows the anatomical position of the selected regions. For each of the 72 locations, the analyzed time series corresponds to the norm of the current dipole vector reconstructed at that location. For each pair of nodes, the geometric distance between the two nodes is computed out of their MNI coordinates.

### Assessment of a functional relationship between *W* and *d*

**Figure 6:**
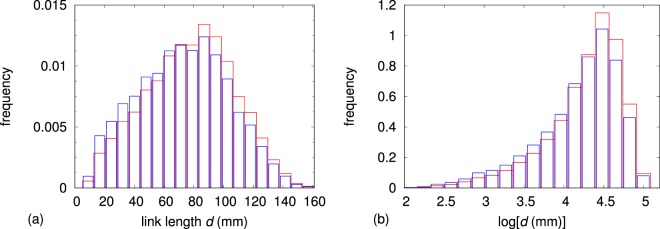
Red lines: histograms of the ∼ 50000 available values of link length *d* (**a**) and its logarithm $\log \,[d({\rm{mm}})]$ (**b**). The number corresponds to 20 subjects and 2556 node pairs for each subject. Each link length actually occurs 3 times, corresponding to the 3 available recordings for each subject. Blue lines: histograms of the ∼ 83000 link lengths *d* (**a**) and its logarithm $\log \,[d({\rm{mm}})]$ (**b**) for which a value of *W* is available.

Figure 6(a) shows the distribution of link lengths evaluated by considering the 20 subjects and the 72 selected nodes for each subject. Two histograms are shown: the first one (red line) refers to the whole set of link lengths while the second one (blue line), which is more shifted to lower values than the previous one, corresponds to those links for which an assessment of the time scale of observability *W* provided a valid result.

We also analyzed, for each single subject, the matching of the histogram of link lengths *d* corresponding to valid values of *W* with the histogram of the link length *d*, also corresponding to valid values of *W*, assessed on the set of all other subjects. The analysis was carried out by using the Kolmogorov–Smirnov test. In all 20 cases, the *p*–value turned out to be close to unity. Link length thus follows the same distribution independently of the subject.

**Figure 7:**
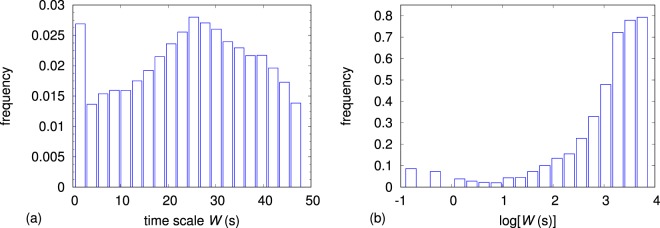
Histograms of the ∼ 83000 available values of time scale *W* (**a**) and its logarithm $\log \,[W({\rm{s}})]$ (**b**).

Figure 7(a) shows the histogram of the ∼ 83000 available values of time scale *W*. While the shape of the histogram shows a maximum in the center of the range as in Fig. 6(a), the presence of a frequency offset of approximately 0.013 in the histogram of Fig. 7(a) forbids the formulation of any linear relationship between *W* and *d*. On the other hand, a linear mapping of the abscissa axes appears to be possible in order to (approximately) overlap the histograms of the logarithm of the two variables *d* and *W*, as it results from the plots of Figs 6(b) and 7(b), and despite *W* being truncated at 48 s, or equivalently $\log \,[W({\rm{s}})]=3.87$, because of experimental reasons. It has also to be noted that no linear mapping can lead to an overlap between *d* and $\log (W)$ and, viceversa, between $\log (d)$ and *W*, thus ruling out the possibility of exponential or logarithmic functional relationships between *d* and *W*.

Consequently, the dependence of the time scale of observability *W* on the link length *d* can be described by means of a power–law curve defined by $W={W}_{0}{(\frac{d}{{d}_{0}})}^{\gamma }$, where the two parameters *W*_0_ and *d*_0_ have the dimension of time and distance, respectively, and the exponent *γ* is dimensionless. To describe the power–law curve, either *W*_0_ or *d*_0_ can be arbitrarily set. The choice was to set *d*_0_ to 75 mm, which approximately corresponds to the average link length (see Fig. 6).

## Data Availability

The authors declare that raw data used in the present work were extracted from the Human Connectome Project public database, which can be accessed at https://db.humanconnectome.org/. Processed data are available upon direct request.
